# Exercise intensity during a minimal equipment circuit session in adults with chronic respiratory disease

**DOI:** 10.1177/14799731261486039

**Published:** 2026-09-04

**Authors:** Christie R. Mellerick, Angela T. Burge, Narelle S. Cox, Anthony May, Janet Bondarenko, Melissa Chong, Anne E. Holland

**Affiliations:** 1 Respiratory Research@Alfred, School of Translational Medicine, 2541 Monash University, Melbourne, VIC, Australia; 2 Institute for Breathing and Sleep, Melbourne, VIC, Australia; 3 Department of Physiotherapy, 5392 Alfred Health, Melbourne, VIC, Australia

**Keywords:** pulmonary rehabilitation, minimal equipment, specialist equipment, exercise training, chronic respiratory disease

## Abstract

**Objective:**

Pulmonary rehabilitation is recommended for people with chronic respiratory disease, yet attendance barriers persist. There is increasing interest in models that may overcome some of these barriers, including minimal equipment exercise training. The capacity of a purely minimal equipment circuit to elicit an acute physiological response sufficient to confer health benefits has not been evaluated. This study assessed whether a minimal equipment circuit session enabled participants to achieve a training intensity of ≥60% $\dot{V}$ O_2_peak.

**Methods:**

People with chronic respiratory disease referred to pulmonary rehabilitation completed a cardiopulmonary exercise test to determine peak oxygen consumption ( $\dot{V}$ O_2_peak), followed by a 60-minute minimal equipment exercise circuit training session. The session included 3 circuits each comprising 7 exercise stations including upper- and lower-limb training. Physiological signs and symptoms were monitored throughout. The primary outcome was the percentage of session spent at or above the desired training intensity (≥60% $\dot{V}$ O_2_peak).

**Results:**

Eighteen participants completed the study (mean (SD) age 67 (11) years). Participants spent a mean 72% (21) of the session at ≥60% $\dot{V}$ O_2_peak, with a median [IQR] of 40[32-47] minutes above this threshold. Fifteen participants (83%) achieved the target intensity for ≥30 minutes. Participants reported positive immediate ratings of confidence, comfort, and enjoyment (5-point Likert scale median 5, IQR 4-5) at the conclusion of the minimal equipment session.

**Conclusion:**

A minimal equipment exercise training session can provide an exercise intensity of ≥60% $\dot{V}$ O_2_peak for >70% of the exercise session in people with chronic respiratory disease. This approach requires further investigation as to whether it may support pulmonary rehabilitation delivery in diverse settings.

## Introduction

Chronic respiratory disease causes distressing symptoms, including breathlessness and fatigue, which can impair a person’s wellbeing, reduce exercise capacity and increase healthcare utilisation.^1–3^ Pulmonary rehabilitation, widely recommended for people with chronic respiratory disease to improve these important symptoms and health-related quality of life,^1,4–6^ is typically delivered as an 8-week outpatient group program with ≥2 supervised exercise sessions per week, following established exercise guidelines to ensure an adequate physiological training stimulus.^7^ Experienced healthcare professionals supervise and titrate individually prescribed exercise training comprising aerobic and resistance exercises, using specialist equipment including treadmills, cycle ergometers, resistance-based machines and free weights, to achieve a positive effect on functional exercise capacity and quality of life.^8^ Despite the well-documented benefits, pulmonary rehabilitation access remains severely constrained. Key barriers including travel and transport issues, limited program availability and capacity, managing comorbid conditions and caring responsibilities prevent many eligible people from participating.^9,10^

There has been increasing interest in alternative models of pulmonary rehabilitation delivery with the aim of overcoming barriers to participation in traditional centre-based program. Minimal equipment program are those that reduce reliance on specialist equipment whilst still delivering core aerobic and resistance training components.^11^ Such programs may make use of readily available, low-cost exercise equipment^12^ such as ground-walking circuits, portable steppers or pedals, hand and ankle weights (up to 5kg) and resistance bands.^13^ Previous studies of minimal equipment exercise training in people with chronic obstructive pulmonary disease (COPD) have demonstrated short-term improvements in functional exercise capacity, commonly assessed using the distance achieved on the 6-minute walk test (6MWT) and health-related quality of life.^14–18^ Recently, Nolan et al.^19^ have reported the largest randomised clinical trial in people with chronic respiratory disease referred for pulmonary rehabilitation and found minimal equipment delivery to be non-inferior to specialist gym-based delivery for exercise capacity, dyspnoea and health-related quality of life. Minimal equipment programs may be able to widen pulmonary rehabilitation delivery options in settings where gym infrastructure, staffing or travel issues are limiting factors for people with chronic respiratory disease.

Guidelines suggest that a training intensity of at least 60% of peak exercise capacity (≥60% $\dot{V}$ O_2_peak) is necessary to elicit a training response within the pulmonary rehabilitation setting.^7^ Although these guidelines are largely derived from continuous endurance-training paradigms, evidence suggests that circuit-based and mixed-modality training can elicit comparable cardiorespiratory responses when the cumulative workload reaches moderate-to-vigorous intensity.^20^ Whether a minimal equipment circuit can elicit an acute physiological response sufficient to confer health benefits has not been evaluated. This is particularly relevant, as achieving moderate-to-vigorous exercise intensity through continuous exercise modalities presents challenges in remotely supervised, minimal equipment settings.

This study aimed to determine the proportion of time (% of session) spent at or above the desired training intensity (≥60% $\dot{V}$ O_2_peak) during a minimal equipment circuit session.

### Study design and methods

This single site acute exercise-intensity characterisation study was conducted at a tertiary referral centre in Melbourne, Australia. Institutional ethics approval was obtained from Alfred Health Human Research Ethics Committee prospectively (Project 100460, Local reference 471/23). All participants provided written informed consent prior to study participation.

### Participants

Adults (age ≥18 years) referred to pulmonary rehabilitations with a confirmed diagnosis of chronic respiratory disease including COPD, interstitial lung disease (ILD), asthma, bronchiectasis or lung cancer were eligible for study inclusion. Exclusion criteria comprised non-ambulatory status, comorbidities which precluded circuit exercise training, supplemental oxygen requirement, inability to provide consent or cognitive impairment precluding understanding of test instructions.

### Study procedures

Participants attended two study visits.

At Visit 1, a symptom-limited incremental cardiopulmonary exercise test (CPET) was performed on an electronically braked cycle ergometer. The CPET included expired breath-by-breath gas analysis of exhaled oxygen (O_2_) and carbon dioxide (CO_2_), ECG monitoring and medical supervision by a trained doctor along with research staff (Accredited Exercise Physiologist or Physiotherapist) as per the international standardised protocol.^21^ The test commenced with 2minutes of unloaded pedalling, followed by a continuous ramp protocol increasing by 10watts per minute until volitional fatigue or symptom limitation. Participants were instructed to pedal at a rate of 50-60rpm throughout the test. Borg rating of perceived exertion (6-20) and modified Borg dyspnoea (0-10) scores were recorded minutely and at test termination.^22^ The $\dot{V}$ O_2_peak was defined as the highest 20-second averaged oxygen consumption measured in the last minute of the test. Following CPET, participants completed a 15-minute familiarisation session during which the minimal equipment circuit was introduced, demonstrated, and individually prescribed, with exercise intensity titrated according to physiological responses and symptom scores in accordance with core pulmonary rehabilitation principles.^8^ Participants were also provided with a familiarisation booklet detailing the minimal equipment exercise stations and circuit layout to take home.

At Visit 2, participants performed a 60-minute minimal equipment exercise circuit session comprising three circuits of seven upper- and lower-limb exercise stations. Exercise bouts ranging from 30-90 seconds based on individual tolerance and were prescribed to achieve modified Borg dyspnoea scale (0-10) score 3-4 in alignment with guidance that this symptom intensity of training corresponds to 60% $\dot{V}$ O_2_peak.^22^ The circuit-based exercises were: 1. Sit to stand; 2. Air boxing; 3. Step up; 4. Stepping out to side with lateral raise; 5. High knees marching on the spot; 6. Wall or bench push up; and 7. Step up (e-Appendix 1). The total session duration was 60 minutes, comprising 45minutes circuit time, including 31.5 minutes of active exercise and 20-second rest periods between exercise bouts. There were 5-minute rest periods between each circuit. Physiological signs and symptoms were monitored throughout the session as per standard procedures. Peak heart rate and nadir peripheral oxygen saturation (SpO_2_, %) were measured using a portable pulse oximeter with ear probe (Nonin PalmSAT 2500A), and symptoms of leg fatigue (Borg rating of perceived exertion, 6-20) and dyspnoea (modified Borg dyspnoea scale, 0-10)^22^ were recorded at the end of each exercise station. Exercise testing and training followed standardised protocols with continuous physiological monitoring. Exercise was ceased for significant symptoms (e.g., chest pain, dizziness, light-headedness) or SpO_2_ ≤88%, consistent with clinical practice.

The MetaMax 3B (Cortex; Leipzig, Germany), an indirect calorimetry system, was used for breath-by-breath measurement of expired gases. MetaMax output including breath-by-breath oxygen uptake ( $\dot{V}$ O_2_) were averaged every 5 seconds. To correctly identify 5-second epochs of exercise data, the start of each exercise interval was “flagged” in the MetaMax 3B software program (MetaSoft) and reviewed against hard copy data record sheets to identify training and rest periods.

Participant demographic information including diagnosis, age, sex, forced expiratory volume in 1 second (FEV_1_, % predicted), forced vital capacity (FVC, % predicted), body-mass index (BMI), comorbidities, smoking history, dyspnoea as measured by the modified Medical Research Council Scale (mMRC) and exercise capacity as measured by the distance achieved on the 6-minute walk test (6MWD) were recorded from baseline pulmonary rehabilitation assessment.

### Outcomes

The primary outcome was the proportion of time spent at or above the desired training intensity (≥60% $\dot{V}$ O_2_peak) determined from the CPET at Visit 1. This pre-defined exercise intensity criterion is consistent with the exercise guidelines stipulated by the American Thoracic Society/European Respiratory Society and American Association of Cardiovascular and Pulmonary Rehabilitation.^7^ An intensity of ≥60% $\dot{V}$ O_2_peak was selected as it represents the lower bound of moderate intensity exercise and approximates the transition into the moderate-to-vigorous intensity domain, balancing efficacy with tolerability in a clinical population. Prescribing exercise at ≥60% $\dot{V}$ O_2_peak also aligns with training intensity achieved when exercise is prescribed using validated symptom scores such as the modified Borg dyspnoea score 3-4,^23^ commonly used in traditional pulmonary rehabilitation settings. The time spent at ≥60% $\dot{V}$ O_2_peak per session was calculated by summing 5-second epochs meeting this intensity threshold across all training bouts and expressed as both minutes and the percentage of total training time. Subsequently, the mean percentage across all participants was calculated. The 20-second rest periods were included in analysis, whereas 5-minute inter-circuit rest periods were excluded because breath-by-breath data were unavailable while participants removed the MetaMax during this period.

Secondary outcomes were symptom and physiological responses during the training session, and program acceptability. Acceptability was assessed for all participants at the completion of the training session across three domains using a 5-point Likert scale (1 strongly disagree to 5 strongly agree). The specific questions were: “I am physically capable of performing this exercise routine” (confidence); “I would be comfortable to perform this exercise routine independently at home” (comfort); and “I enjoyed completing these exercises” (program enjoyment).

### Statistical analysis

Variables demonstrating an approximately normal distribution (normality determined by visual inspection) are presented as mean (standard deviation, SD), while variables used to estimate precision around key outcomes are additionally reported as mean 95% confidence interval (95% CI). Non-normally distributed variables are presented as median [interquartile range (IQR)]. The primary outcome (% of session time spent ≥60% VO_2_peak) was analysed descriptively, specific to the study aim. Descriptive statistics were used for categorical data, expressed as n (%). Analyses were performed using SPSS (v28.0, IBM, Armonk, NY, USA).

### Sample size

A total of 18 participants were required for this study. Measures of $\dot{V}$ O_2_ during maximal and sub-maximal exercise are highly reproducible in people with chronic lung disease, with test-to-test variability of 5-10% of $\dot{V}$ O_2_peak^.24^ To detect a difference of ≥10% from the expected mean value of 60% $\dot{V}$ O_2_peak, representing a difference that exceeds measurement error, a total of 18 participants were required. This calculation was based on an assumed standard deviation of 15% for submaximal $\dot{V}$ O_2_ responses in people with chronic lung disease.^25^

## Results

**Figure 1.**
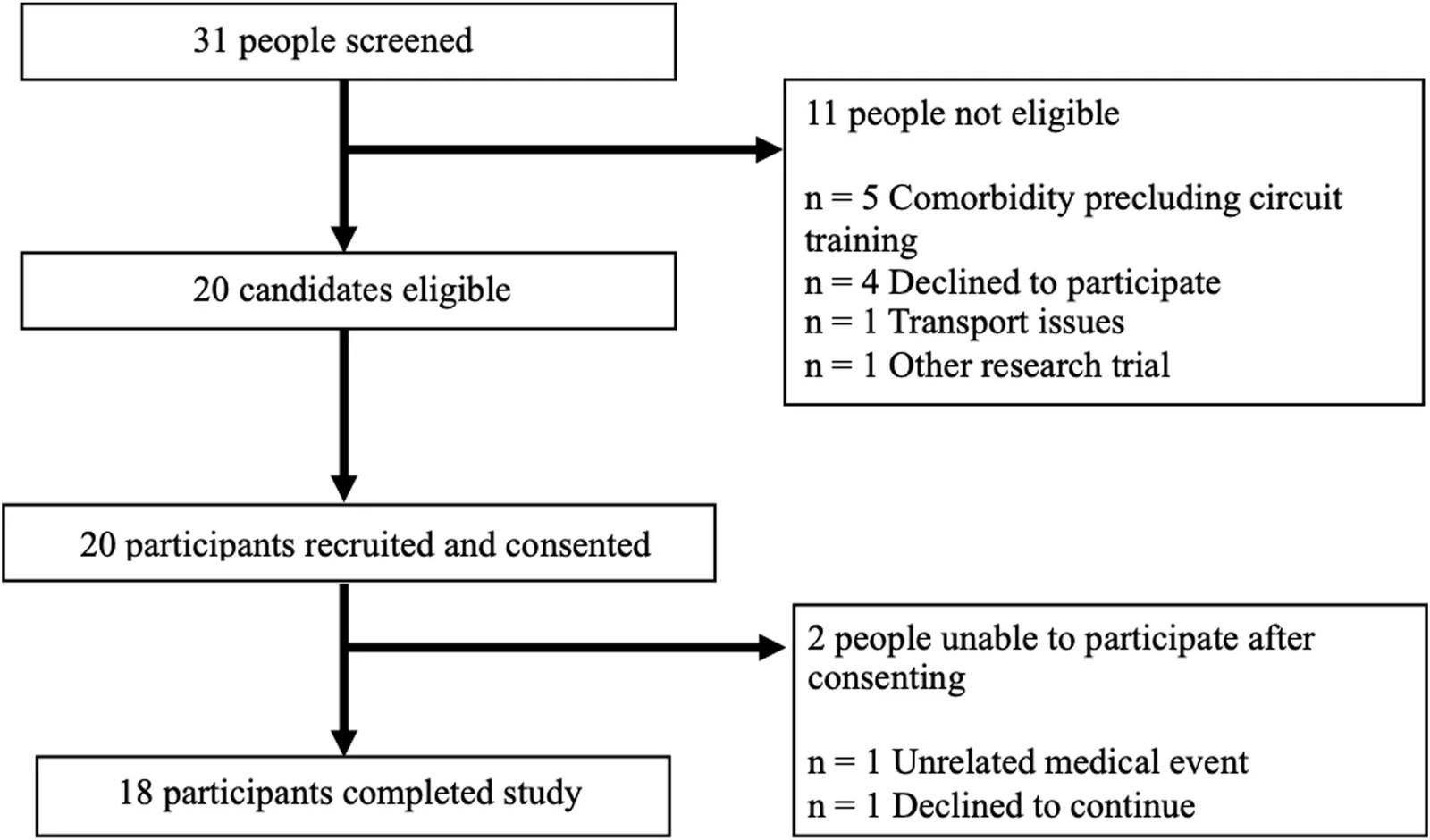
Participant flow.

20 participants were recruited between November 2023 and August 2024, with 18 completing all study procedures. Two participants did not proceed with the study following consent (Figure 1). The mean age of participants was 67 (SD 11) years, and majority of participants had a diagnosis of COPD or asthma (33% each), followed by bronchiectasis (22%), with ILD and lung cancer each accounting for 6%. Participants reported mild symptoms of shortness of breath (mMRC), and functional exercise capacity was generally high, with the highest 6MWD observed in participants with bronchiectasis (mean 586 m, SD113) (Table 1).

**Table 1.** Participant characteristics.

​		n=18
Diagnosis, *n (%)*	COPD	6 (33)
	Asthma	6 (33)
	Bronchiectasis	4 (22)
	ILD	1 (6)
	Lung cancer	1 (6)
Female, *n (%)*	​	14 (78)
Age, *years*	​	67 (11)
BMI, *kg/m*^*2*^	​	26 (5)
FEV_1,_ *% predicted* (n=13) median [IQR]	​	74 [65 to 88]
FVC, *% predicted* (n=16) median [IQR]	​	84 [77 to 97]
Smoking status, *n (%)*	Current	0 (0)
	Ex-smoker	12 (67)
	Non-smoker	6 (33)
Pack years (n=10), median [IQR]	​	30 [11, 56]
Modified Medical Research Council score *0/1/2/3/4* (n=16)	​	5/8/3/0/0
6MWD, *meters*	​	509 (86)
$\dot{V}$ O_2_peak, *L/min*	​	1.3 (0.4)

Data are mean (SD) unless indicated.

BMI = body mass index, COPD = chronic obstructive pulmonary disease, FEV_1_ = air forcefully exhaled from lungs in 1 second, ILD = interstitial lung disease, IQR = interquartile range, SD = standard deviation, 6MWD = 6-minute walk distance.

**Figure 2.**
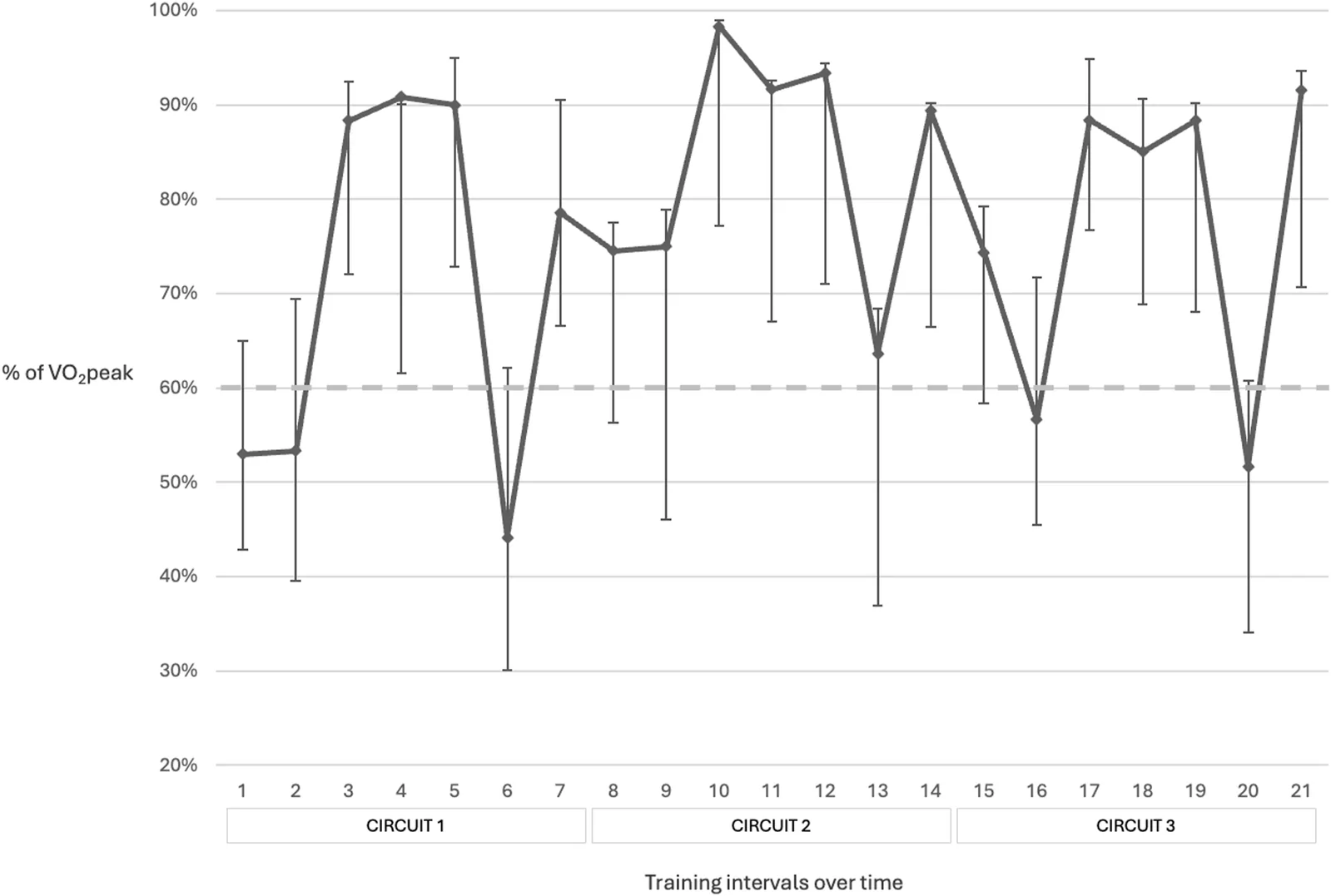
Mean % of $\dot{V}$ O_2_peak per exercise station during minimal equipment session. Exercise training data are represented by mean $\dot{V}$ O_2_% (95% CI). The centre diamond points represent the mean $\dot{V}$ O_2_%, with the top and bottom points of the lines indicating the upper and lower quartiles. The x-axis represents the training session and corresponding exercise time points. Legend for exercise stations: Sit to stand = 1, 8, 15, Air boxing = 2, 9, 16, Step up = 3, 6, 10, 13, 17, 20, Stepping out to side with lateral raise = 4, 11, 18, High knees marching on spot = 5, 12, 19, Wall or bench push up = 7, 14, 21.

Mean 72% (95% CI 60 – 81%) of the minimal equipment circuit session time was spent at intensity ≥60% $\dot{V}$ O_2_peak (Figure 2, Table 2). Median session time spent ≥60% $\dot{V}$ O_2_peak was 40 [IQR 32 – 47] minutes with 15 out of 18 participants (83%) achieving the desired training intensity for at least 30 minutes (Figure 2, Table 2). Exploratory descriptive analyses showed similar median proportions of time spent at ≥60% $\dot{V}$ O_2_peak across the major diagnostic groups: COPD 77% [IQR 68 – 90]; Bronchiectasis 69% [IQR 49 – 88]; and Asthma 73% [IQR 50 – 88] [Supplemental material, Table 3]. No formal statistical comparisons were undertaken due to the small sample size.

**Table 2.** Physiological and symptom response during circuit session.

​	Exercise stations						
	1. Sit to stand	2. Air boxing	3. Step up	4. Side-step with lateral raise	5. High knees marching on spot	6. Wall push-up	7. Step up
% $\dot{V}$ O_2_peak	64 (60 – 70)	58 (50 – 66)	85 (80 – 91)	80 (73 – 87)	82 (75 – 89)	49 (41 – 58)	80 (73 – 86)
Heart Rate, *bpm*	108 (104 – 112)	111 (107 – 115)	114 (110 – 118)	113 (109 – 118)	114 (111 – 118)	110 (106 – 113)	117 (113 – 121)
SpO_2_, *%*	97 (96 – 97)	96 (95 – 97)	96 (95 – 97)	97 (96 – 97)	96 (96 – 97)	98 (97 – 99)	96 (95 – 97)
Borg rating of Perceived Exertion (6-20)	12 (12 – 13)	12 (12 – 13)	13 (12 – 14)	13 (12 – 14)	13 (13 – 14)	13 (12 – 14)	13 (12 – 14)
Borg rating of Perceived Dyspnoea (0-10)	3 (3 – 4)	3 (3 – 4)	4 (3 – 4)	4 (3 – 4)	4 (3 – 4)	4 (3 – 4)	4 (3 – 4)

Data are mean (95% CI).

Bpm = beats per minute, CI = confidence interval, SpO_2_ (%) = peripheral oxygen saturation percentage, $\dot{V}$ O_2_peak = peak oxygen consumption.

**Figure 3.**
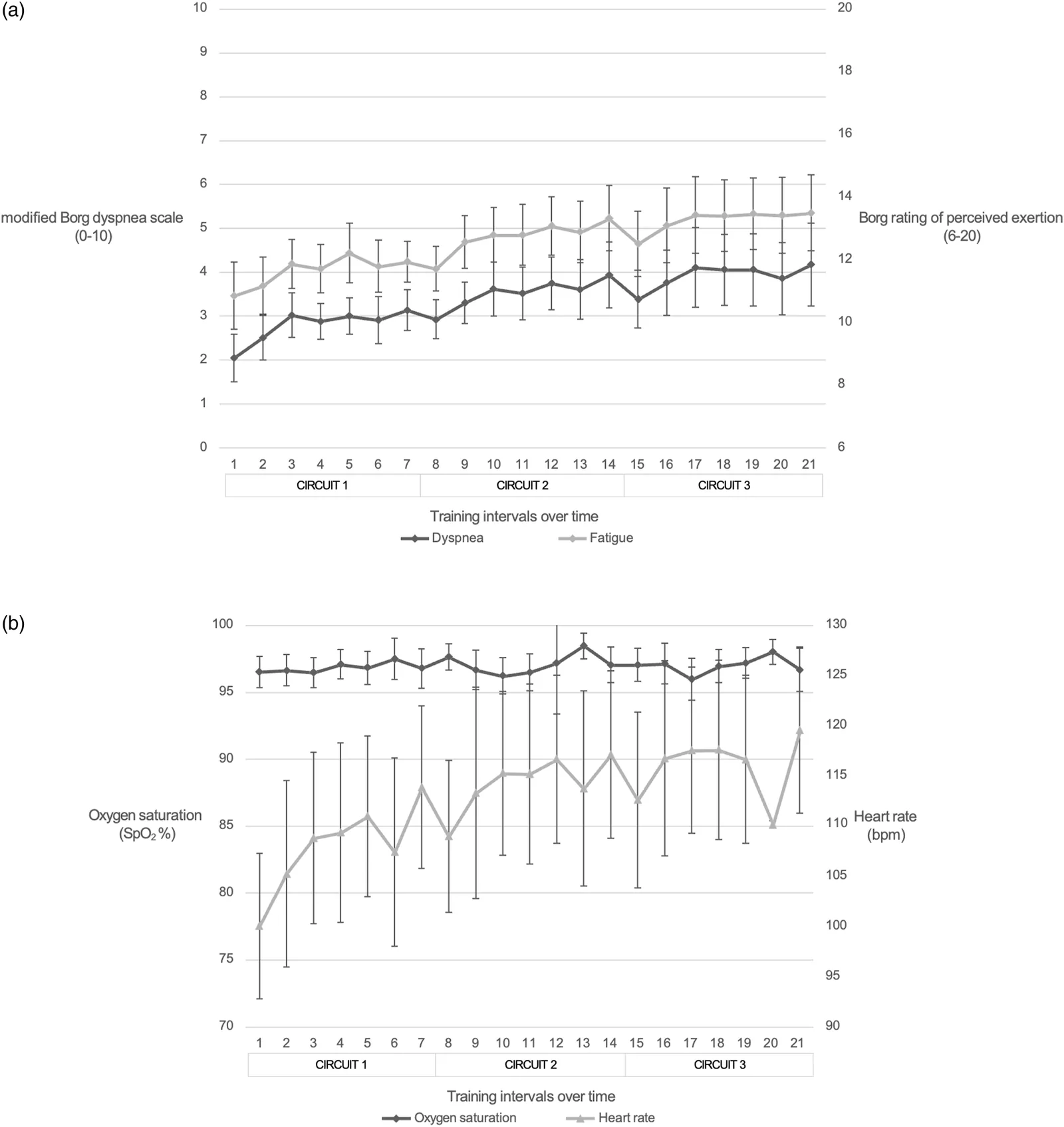
(a) Borg symptom response during training session. The data are represented by mean (95% CI). The centre diamond point represents the mean Borg dyspnoea and fatigue values. (b) Physiological response during training session. The data are represented by mean (95% CI). The centre diamond point represents the mean HR, SpO_2_. The top and bottom points of the lines show the upper and lower quartile values. The x-axis represents the training session and corresponding exercise time points.

Physiological and symptom outcomes showed little apparent variation across the session [Figure 3(a) and (b)] with mean Borg rating of perceived exertion score 13 (95% CI 12 – 14) and modified Borg dyspnoea score 4 (95% CI 3 – 4). Missing data due to technical issues or incomplete physiological recordings were assumed to be completely at random and excluded from analyses for the affected variable only. Missing values were observed for SpO_2_ (n=32, 4%), heart rate (n=4, 1%), modified Borg dyspnoea score (n=10, 1%), and Borg rating of perceived exertion (n=10, 1%). The interval training is comprised of moderate-to-vigorous intensity exercises as outlined in Table 2.

The mean percentage of $\dot{V}$ O_2_peak showed minimal variation across training bouts, with the highest values recorded in training bouts 1 to 14 (Figure 2). Across individual exercises, the highest percentage of $\dot{V}$ O_2_peak was demonstrated for step ups (exercise 3, exercise 7) mean 85% (95% CI 80 – 91), mean 80% (95% CI 73 – 86), and high knees marching on the spot (exercise 5), mean 82% (95% CI 75 – 89), (Table 2). Wall or bench push up (exercise 6) exhibited the most variation in percentage of $\dot{V}$ O_2_peak across participants, mean 49% (95% CI 41 – 58).

**Figure 4.**
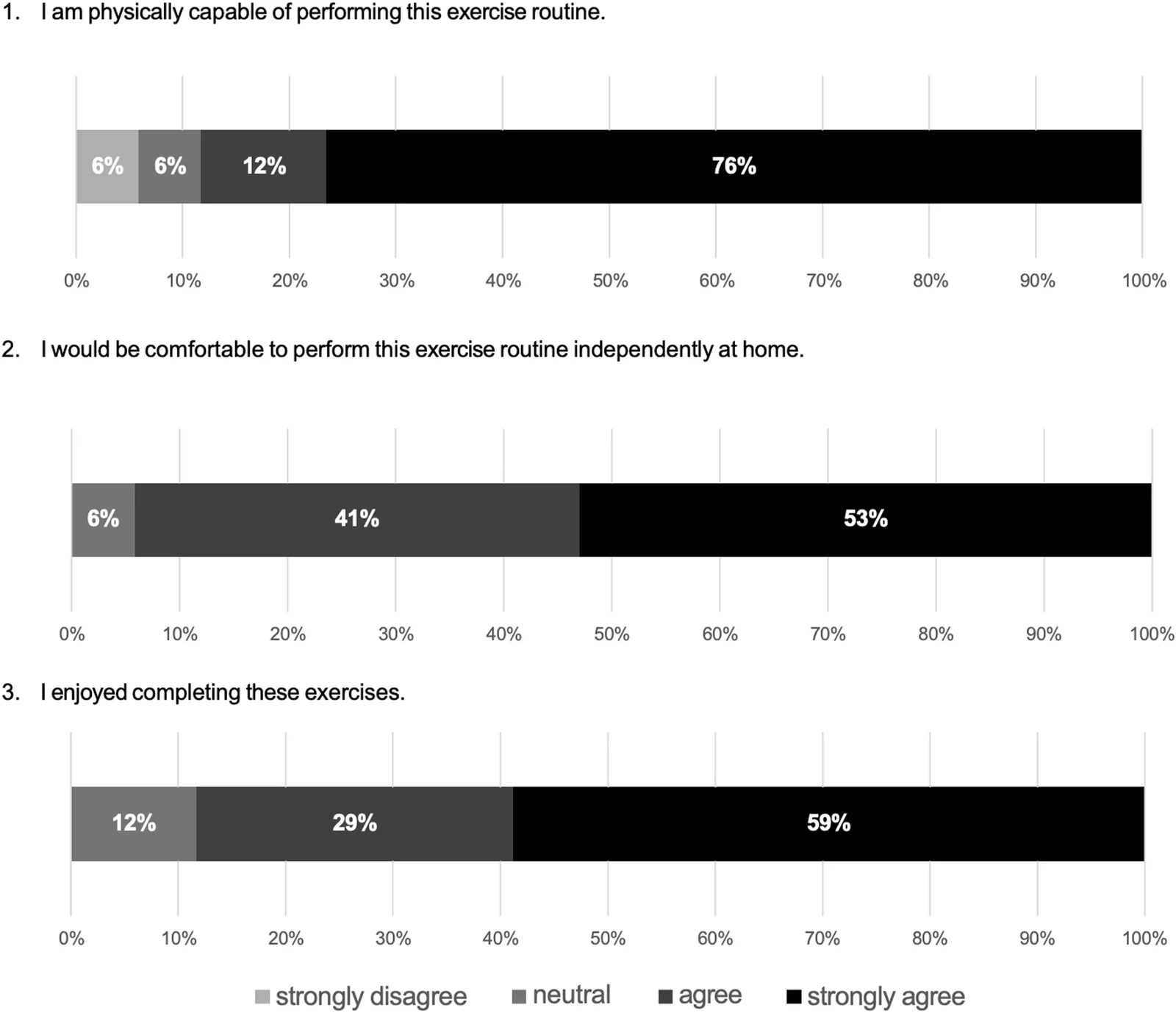
Acceptability of a minimal equipment exercise session assessed using a 5-point Likert scale. The data are represented by three stacked bar charts, the shade in the legend correlates to responses around acceptability of the training session in participants, as represented by %.

Participants reported high post-session confidence, comfort and enjoyment median 5 [IQR 4 – 5] (Figure 4).

## Discussion

This study demonstrates that a single minimal equipment circuit session can elicit a moderate-to-vigorous exercise response in people with chronic respiratory disease. Unlike previous studies of minimal equipment training in the pulmonary rehabilitation setting, which have not assessed exercise intensity,^11^ this work uniquely quantifies the physiological intensity achieved during a single session minimal equipment circuit, demonstrating that over 80% of participants attained ≥30 minutes of moderate-to-vigorous intensity exercise (≥60% $\dot{V}$ O_2_peak). If performed 3-5 days per week, this would achieve the aerobic training dose recommended in pulmonary rehabilitation guidelines (20-60minutes of moderate-to-vigorous intensity on 3-5 days per week).^23^Achieving moderate intensity exercise (≥60% $\dot{V}$ O_2_peak) is acknowledged as necessary to drive physiological adaptations,^23^ and whilst prior interval and circuit based pulmonary rehabilitation studies have demonstrated comparable outcomes to continuous training, they have typically relied on specialist equipment or walking-based aerobic stimulus.^20^ Minimal equipment models have demonstrated promise in improving accessibility but often lack precise control on exercise intensity, particularly in remote or unsupervised settings. Consistent reporting of Borg symptom scores within the target range^23^ throughout the training session provides supportive evidence that a minimal equipment circuit may be an appropriate modality for delivering a pulmonary rehabilitation exercise stimulus. These findings suggest that a minimal equipment circuit has the potential to deliver an aerobic training stimulus comparable to that prescribed in traditional pulmonary rehabilitation.

In our study, variations in $\dot{V}$ O_2_peak were evident throughout the session depending on the specific exercise performed. Although physiological demand varied between the individual circuit-based exercises, with exercise 6 (wall or bench push up) consistently demonstrating the largest variation in $\dot{V}$ O_2_ across participants, the target intensity was generally maintained across the session. The exercise selection and sequencing were designed to facilitate recovery by alternating aerobic tasks with isolated functional movements, to achieve modified Borg dyspnoea scale (0-10) score 3-4 in alignment with guidance that this symptom intensity of training corresponds to 60% $\dot{V}$ O_2_peak.^23,26^ To optimise consistency of exercise intensity for future projects adopting this model of exercise training, the inclusion of exercise 6 (wall or bench push up) needs to be reviewed. This emphasizes the importance of evaluating training intensity and appropriate exercise selection in consideration of the physiological demand in the context of pulmonary rehabilitation. Although progression of exercise training loads over time is also an essential component of pulmonary rehabilitation,^8^ our study did not address this in the single-session design. This study provides preliminary physiological data that may help inform future prescription and progression of minimal equipment studies.

Participants reported the minimal equipment circuit session was acceptable in terms of confidence, comfort and enjoyment. These factors are important considerations in the early evaluation of a novel training model, as they may influence engagement and, ultimately, program completion. Pulmonary rehabilitation program acceptability has been shown to be a factor in achieving program completion,^27^ which in turn is necessary for achieving clinical benefits of rehabilitation and reduction in subsequent healthcare utilisation.^15,28^ Acceptability of minimal equipment programs delivered in a home-based setting may support long-term program adherence.^29^ Given the multitude of documented barriers that impact pulmonary rehabilitation uptake, participation and completion,^30^ broadening the scope of patient-centred approaches to rehabilitation delivery, including those with minimal equipment requirements and ability to be delivered in non-standard settings, may help to create opportunities for more people to access pulmonary rehabilitation across a variety of healthcare contexts.

Key strengths of this study include the rigorous direct measurement of exercise intensity during minimal equipment training. A limitation of this study was the small sample size; however, the study achieved the target sample size for the primary outcome. Additionally, the sample comprised participants with mixed diagnoses who generally had mild disease severity, symptom burden and relatively preserved functional capacity, as evidenced by their baseline mMRC, 6MWD and $\dot{V}$ O_2_peak. As such, the generalisability of these findings to people with more severe lung disease where % $\dot{V}$ O_2_peak required to perform circuit exercises may be higher,^31,32^ remains uncertain. Variability in exercise demands (Figure 2) highlights the importance of individualised prescription and titration to achieve the desired training intensity. While symptom-guided, standardised instructions may not have maximised the physiological stimulus at each exercise station; refining exercise-cues, such as encouraging a faster movement tempo while maintaining correct technique, may increase the proportion of time spent within the target intensity range in future implementations. While time ≥60% $\dot{V}$ O_2_peak was used in this study to quantify aerobic intensity consistent with pulmonary rehabilitation guidelines, functional and resistance-based tasks likely impose additional neuromuscular, neuromotor and symptom related demands relevant to daily function and rehabilitation outcomes in people with chronic respiratory disease. Accordingly, time spent above the $\dot{V}$ O_2_ threshold represents only one marker of exercise intensity rather than a comprehensive measure of the overall training stimulus. Although an a priori target for the proportion of session time spent at ≥60% $\dot{V}$ O_2_peak was not specified, participants spent, on average, 70% of the 45-minute session above this threshold. A further limitation is that training thresholds were derived from cycle-based CPET, which provides a different training stimulus (seated) than does minimal equipment training (upright exercise) despite the use of gold-standard CPET procedures. The different physiological demands of seated versus upright exercise could affect estimates of training intensity, consistent with evidence demonstrating higher $\dot{V}$ O_2_ peak during 6-minute walk test than cycle-based CPET in individuals with chronic conditions.^33–35^ Future studies should consider modality-specific assessments to more accurately characterise training intensity, given that exercise intensity is an important determinant of training outcomes.^36^

## Conclusion

A minimal equipment circuit can provide an exercise intensity of ≥60% $\dot{V}$ O_2_peak for >70% of the exercise session in people with chronic respiratory disease. Whether the demonstrated minimal equipment circuit can support access to pulmonary rehabilitation requires investigation.

## Relevance

This study has demonstrated that a minimal equipment circuit achieves the target exercise intensity (≥60% $\dot{V}$ O_2_peak) in people with chronic respiratory disease, supporting further investigation of this training modality in pulmonary rehabilitation.

Minimal equipment circuit training may offer a practical, lower cost and more accessible alternative to traditional pulmonary rehabilitation. The present study provides preliminary evidence that minimal equipment circuit training can elicit an acute physiological stimulus at the target training intensity, supporting further investigation into its potential to address key barriers to program access and participation and expand pulmonary rehabilitation delivery beyond conventional centre-based settings.

## Supplemental material

Supplemental material - Exercise intensity during a minimal equipment circuit session in adults with chronic respiratory disease Supplemental material for Exercise intensity during a minimal equipment circuit session in adults with chronic respiratory disease by Christie R Mellerick, Angela T Burge, Narelle S Cox, Anthony May, Janet Bondarenko, Melissa Chong and Anne E Holland in Chronic Respiratory Disease.
