# Supplementary material for: Exercise intensity during a minimal equipment circuit session in adults with chronic respiratory disease
Source: Chron Respir Dis. 2026 Sep 4;23:14799731261486039. doi: 10.1177/14799731261486039 (PMC13547803; doi:10.1177/14799731261486039)
Supplement: Supplementary file 1 [file sj-pdf-1-crd-10.1177_14799731261486039.pdf]

Table 3. Subgroup descriptive analysis by diagnostic group.

Diagnosis	n=16	% session spent at ≥60%$\dot{V}O_2$peak	Borg rating of Perceived Exertion (6-20)	Borg rating of Perceived Dyspnoea (0-10)
COPD	6	77 [68 – 90]	12 (12 – 13)	4 (3 – 4)
Asthma	6	73 [50 – 88]	13 (12 – 15)	4 (3 – 5)
Bronchiectasis	4	69 [49 – 88]	12 (9 – 14)	3 (2 – 4)

Data are median [IQR], mean (95% CI).

CI = confidence interval, $\dot{V}O_2$peak = peak oxygen consumption

*ILD (n=1) and Lung Cancer (n=1) excluded due to small number.

e-Appendix 1

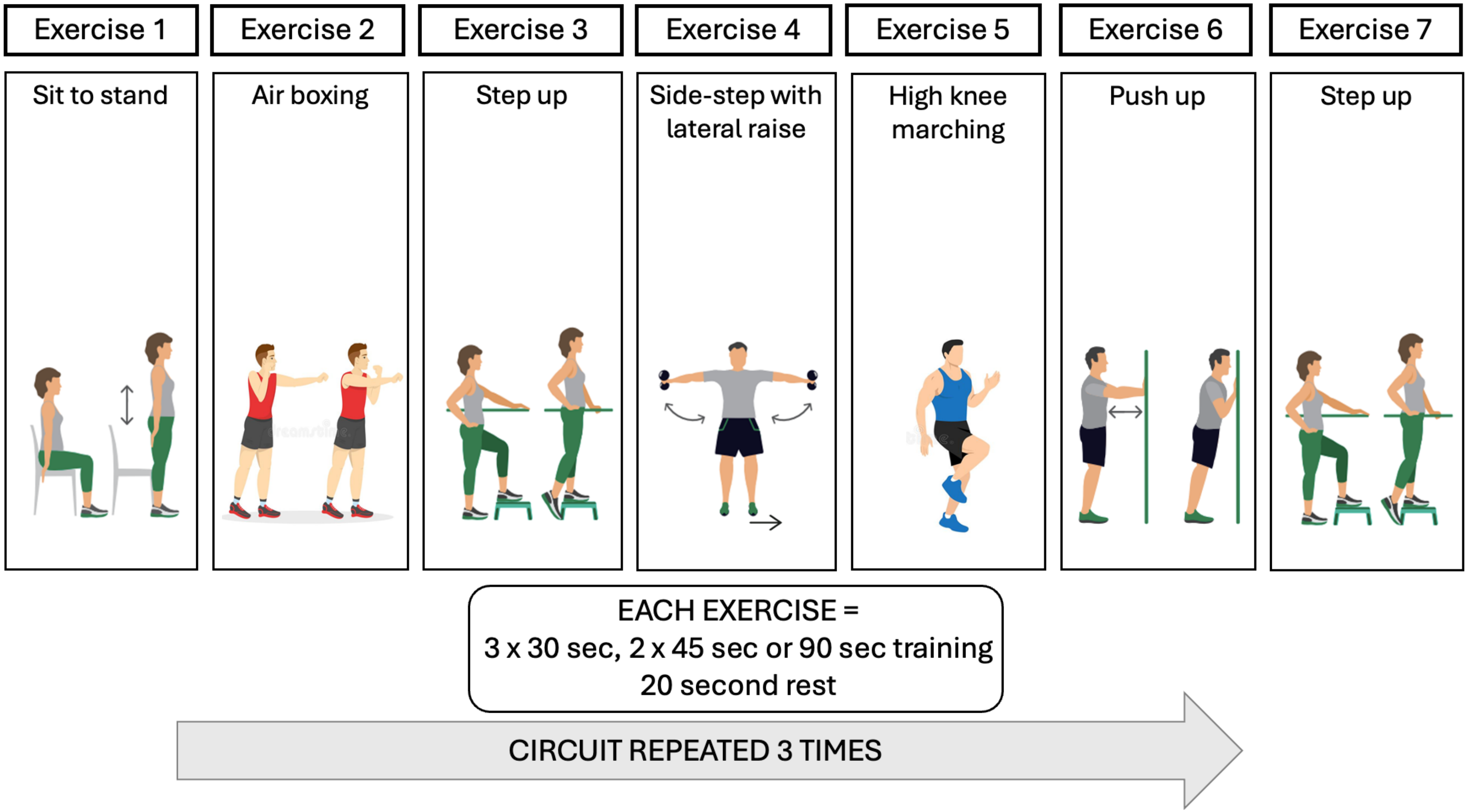
Exercise 1
Sit to stand
Exercise 2
Air boxing
Exercise 3
Step up
Exercise 4
Side-step with lateral raise
Exercise 5
High knee marching
Exercise 6
Push up
Exercise 7
Step up
EACH EXERCISE =
3 x 30 sec, 2 x 45 sec or 90 sec training
20 second rest
CIRCUIT REPEATED 3 TIMES
